# A Novel Approach of Ultrasound-Guided Transforaminal Cervical Nerve Root Injection With Fluoroscopic Validation: A Case Report and Technical Illustration

**DOI:** 10.7759/cureus.74937

**Published:** 2024-12-01

**Authors:** Tsung Ju Wu, Chih-Wei Lee, King Hei Stanley Lam, Chen-Yu Hung

**Affiliations:** 1 Regenerative Medicine, Reboot Clinics, Changhua, TWN; 2 Physical Medicine and Rehabilitation, Changhua Christian Hospital, Changhua, TWN; 3 Radiology, Changhua Christian Hospital, Changhua, TWN; 4 Faculty of Medicine, The University of Hong Kong, Hong kong, HKG; 5 The Board of Clinical Research, The Hong Kong Institute of Musculoskeletal Medicine, Kowloon, HKG; 6 Faculty of Medicine, The Chinese University of Hong Kong, New Territory, HKG; 7 Pain and Rehabilitation, Regen Clinic, Taipei, TWN

**Keywords:** cervical, pain, radiculopathy, transforaminal injection, ultrasound

## Abstract

Cervical radiculopathies are commonly treated with nerve root injections. This study presents a novel ultrasound (US)-guided cervical transforaminal injection technique using a curved transducer to enhance visualization of the anterior wall of the superior articular process and achieve successful epidural spread of injection. A 56-year-old patient with chronic C6 radiculopathy was treated using our US-guided approach with a combination of 5% dextrose and vitamin B12, leading to significant improvement in symptoms. Our approach demonstrated accurate needle placement and effective injectate spread to the epidural space while minimizing the risk of vascular injury. This technique offers reduced fluoroscopy usage time, potentially reducing radiation exposure and improving therapeutic outcomes in the management of cervical radiculopathy.

## Introduction

Cervical radiculopathies, though less common than their lumbar counterparts, have an incidence rate of approximately 85 cases per 100,000 people [1]. This condition arises when a nerve root in the neuroforamen becomes compressed or irritated. Compression on the anterior aspect is primarily attributed to protruding discs and osteophytes in the uncovertebral region, while posterior compression often results from hypertrophied superior articular processes and/or the ligamentum flavum. When a nerve root is compressed or irritated, it can lead to symptoms such as pain, numbness, tingling, and weakness, which may radiate beyond the neck into the arm, chest, shoulder, and upper back, depending on the specific cervical nerve root affected. Over time, these symptoms can exacerbate, potentially leading to a decline in mental health, physical functioning, and social participation [2].

The use of extraforaminal selective nerve root blocks has gained popularity with the growing application of ultrasound (US) technology [3]. Various injectates are commonly employed, including local anesthetics [4], non-particulate steroids [5], vitamin B12 [6], and 5% dextrose [7]. This procedure involves identifying the anterior and posterior tubercles of the transverse process using a linear US transducer, allowing for the circumferential spreading of injectate around the selective nerve root within the intertubercular groove of the corresponding transverse process outside the intervertebral foramen [8].

The main advantage of the US-guided procedure is that it is free from radiation exposure. However, the retrograde spread of injectate from the extraforaminal selective nerve root injection to the transforaminal area is typically limited, with most of the injectate spreading downstream toward the interscalene region [9]. The advantage of the fluoroscopic approach is its ability to clearly identify bony landmarks. However, irritation of the nerve root during needle advancement is inevitable due to a lack of real-time soft tissue imaging. Consequently, the fluoroscopic approach to transforaminal blocks remains the preferred method for clinicians aiming to treat cervical radicular symptoms and achieve epidural spread of the injectate. In fluoroscopy-guided transforaminal injections, the posterior portion of the foramen, formed by the anterior wall of the superior articular process, serves as the key anatomical landmark [4].

Despite the availability of established techniques for fluoroscopy-guided transforaminal injections, there is currently no literature detailing how to identify the anterior wall of the superior articular process under US guidance. In this article, we present a novel technique using a curved probe to perform cervical transforaminal injections by accurately identifying this landmark. The research was conducted by adhering to the principles outlined in the Declaration of Helsinki. Written informed consent was obtained from the patient for the publication of case details and any accompanying images. The institutional review board waived approval for this case report, as it contains no identifiable information.

## Case presentation

A 56-year-old female presented with chronic neck pain (VAS 3-4) and left arm/forearm numbness for several years. A cervical spine MRI indicated a degenerative C5/6 disc, leading to left C6 nerve root compression. Despite undergoing multiple sessions of cervical traction, her symptoms showed limited improvement. She had also received an intradiscal platelet-rich plasma injection at the C5/6 level, which led to fair improvement in axial neck pain, but the left arm/forearm numbness persisted. On physical examination, her neck range of motion was restricted due to pain and numbness during left rotation (45 degrees) and left side bending (45 degrees). Spurling's and upper limb tension tests were positive on the left side. Neurological examination revealed a mildly decreased pin-prick sensation over the left C6 dermatome, with muscle strength remaining intact. Under the impression of left C6 radiculopathy caused by intervertebral neuroforamen narrowing, we proceeded with a left C6 root transforaminal injection, using D5W and vitamin B12 to relieve the nerve root compression.

The procedure was performed on an outpatient basis. A pillow was placed under the patient's upper back to facilitate slight cervical extension. With the patient's head externally rotated 30-40 degrees away from the procedure site, she was positioned supine. The frontal neck area was then adequately disinfected with chlorhexidine, and aseptic draping was applied. A curved US transducer (C2-9-D, GE Healthcare, Chicago, IL) was utilized in our US-guided procedure for better penetration to visualize the anterior wall of the superior articular process within the intervertebral neuroforamen.

**Figure 1:**
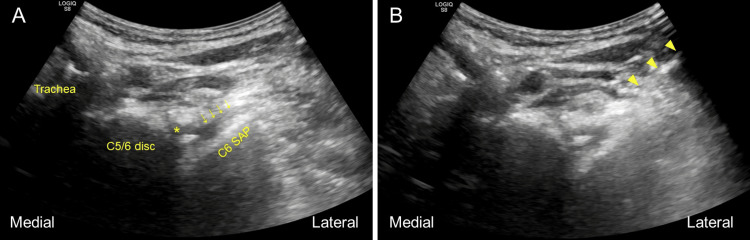
(A) C6 superior articular process was identified as a hyperechoic slope in axial plane using a curved ultrasound transducer. Vertebral artery (asterisk) could be identified anterior to C6 root (arrows). (B) A needle (arrowheads) was inserted with an in-plane approach, parallel with the slope of superior articular process

The C6 vertebral level was identified based on two key anatomical landmarks. First, it is the most caudal cervical vertebra that has both anterior and posterior tubercles on the transverse process, in contrast to C7, which only has the posterior tubercle. Second, the vertebral artery enters the transverse foramen at the C6 level, while at C7 it exits the foramen [8]. After confirming the C6 level, the superior articular process of C6 was identified (Figure 1A), located cranial to the transverse process. Following local anesthesia with 1% lidocaine to numb the skin, a 23G 70-mm hypodermic needle was inserted in a posterior-to-anterior direction using an in-plane approach, aiming parallel to the slope of the superior articular process and inside the neuroforamen (Figure 1B). The needle was advanced until it reached the center of the superior articular process, ensuring it did not pass beyond this point to minimize the risk of dural puncture.

**Figure 2:**
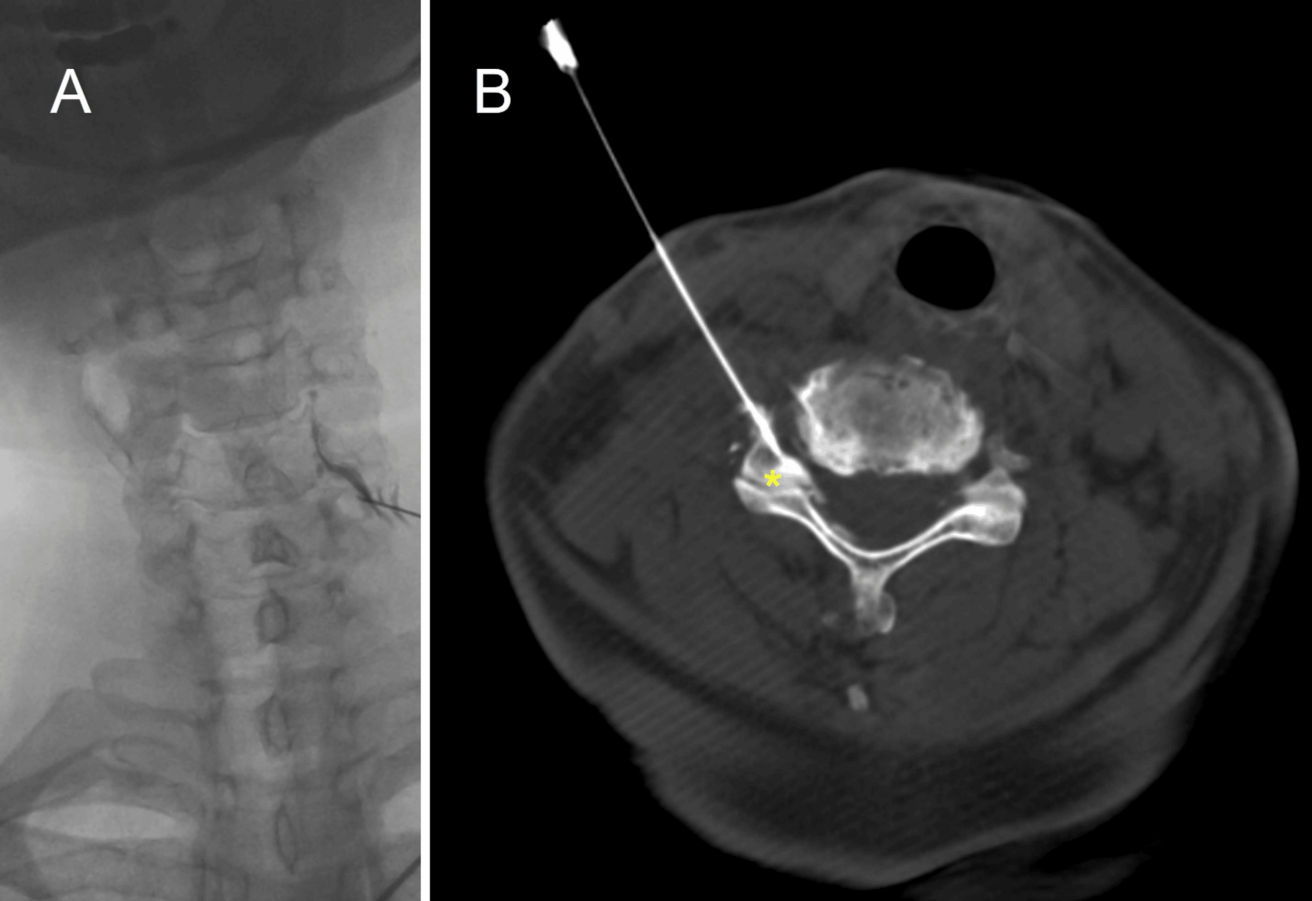
(A) Anteroposterior image of the digital subtraction angiography revealed the spread pattern of the injected contrast media from the center of superior articular process to epidural space. The trajectory of the needle was confirmed with cone beam CT, which showed that the needle was advanced parallel with the slope of superior articular process (asterisk) CT: computed tomography

Digital subtraction angiography (Artis Zee Biplane, Siemens Healthineers, Erlangen, Germany) was used to obtain an optimal oblique view of the intervertebral foramen [10]. Contrast media (Visipaque 320; GE Healthcare) was injected, and the distribution of the contrast was observed in the foramen, nerve root, and epidural space. Anteroposterior images were later obtained to confirm the spread pattern of the contrast (Figure 2A). The needle trajectory was further confirmed using cone beam CT (Figure 2B). A test dose of 1 ml of 1% lidocaine was injected, and the patient was observed for one to two minutes for any concerning signs or symptoms, including mid-neck or contralateral arm pain, metallic taste, dizziness, tachycardia, full-body paresthesia, auditory changes, slurred speech, or motor ataxia [11]. After confirming the absence of adverse reactions, the final injectate (5% dextrose mixed with vitamin B12, methylcobalamin) was administered. The procedure was completed smoothly, and the patient reported significant improvement in the left arm/forearm numbness two weeks after the injection. Her neck range of motion also improved, with both left rotation and left side bending reaching 70 degrees, without pain or numbness.

## Discussion

Unlike selective nerve root injections, transforaminal root injections aim for a more proximal needle placement. This is typically achieved under fluoroscopy guidance, which allows for precise needle placement near the intervertebral neuroforamen, with clear visualization of bony structures. The success of the injection is indicated by the epidural spread of contrast [12]. However, sometimes the patients may feel an electric shock sensation when the needle accidentally touches the nerve root before reaching the neuroforamen [13]. This could be attributed to the lack of visibility of soft tissues in the fluoroscopic approach. Also, to avoid cannulating the vertebral artery and vein, the landmark of needle placement would be the posterior portion of the neuroforamen [14].

Recently, US has been shown to enhance the safety and effectiveness of cervical radiculopathy treatment by providing real-time visualization of soft tissues, critical vessels, and needle advancement throughout the procedure. The most common US-guided technique for cervical nerve root injection targets the nerve root between the anterior and posterior tubercles of the transverse process, which is still some distance from the true neuroforamen [8]. Ma et al. reported that only 9.52% of US-guided cervical root injections at this level led to retrograde spread into the epidural space [15]. Wu et al. recently introduced a novel US-guided in-plane approach that targets the nerve root closer to the intervertebral foramen [16]. In this method, the patient was positioned on their side, with a linear transducer placed transversely on the posterolateral side of the neck, slightly cranial to the junction of the transverse process and the articular pillar at the targeted level. This transducer position allowed visualization of the nerve root as it began to traverse into the neuroforamen, and the needle was directed to the space between the nerve root and the articular pillar. Fluoroscopic imaging revealed that 81% of patients treated using this approach had retrograde spread into epidural space.

In our approach, we utilized a similar anterolateral technique but employed a curved transducer, which provided better US penetration and facilitated the visualization of the anterior wall of the superior articular process. Unlike the US images from Wu et al., where the portion of the nerve root within the intervertebral foramen was obscured by the shadow of the articular pillar, our technique allowed clear visualization of neuroforamen, enabling more proximal needle placement. Also, we were able to check the needle position using fluoroscopy without turning the patient to a supine position. As the needle advanced along the slope of the superior articular process (posterior portion of the neuroforamen), we were able to avoid cannulating the vertebral vessels, which lay on the anterior aspect of the neuroforamen [14].

**Figure 3:**
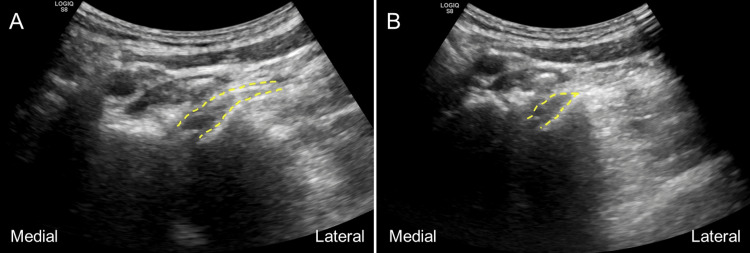
(A) Identify the long axis of the targeted cervical nerve root (dash lines) first using a curved ultrasound transducer. (B) Then, slightly pivot the transducer to the oblique axis of the cervical nerve root, where the root on the top of superior articular process still visible, to avoid the needle puncturing the exiting nerve root The images belong to the patient in this case report

Several key aspects of our US-guided cervical nerve root injection technique should be highlighted. Firstly, by performing a scouting scan of the nerve root using US, we can plan the injection trajectory obliquely to the long axis of the cervical nerve root, thereby minimizing the risk of nerve root puncture (Figures 3A, 3B). Second, during the procedure, we utilized a hydrodissection technique while advancing the needle. This approach helped reduce discomfort by minimizing irritation of superficial tissues along the needle's path. Third, while a "safe zone" for cervical transforaminal injections has been suggested in the posterior-superior quadrant of the foramen [17], it is important to acknowledge that no area is entirely free from risk. Huntoon has highlighted that even in this zone, there remains a 1% risk of injuring the anterior spinal artery [18].

Additionally, Lagemann et al. have reported that up to 45% of injections in the cervical neuroforamen could inadvertently become intravascular, often necessitating needle repositioning [19]. Therefore, fluoroscopic confirmation of needle placement and close monitoring for potential intravascular injections are essential. In particular, the use of particulate steroids raises additional concerns, as these agents have the potential to cause catastrophic neurological sequelae, including brain and spinal cord infarctions if inadvertently taken up by the vasculature [20]. To mitigate these risks, we incorporated digital subtraction angiography and an anesthetic test to confirm needle safety. Furthermore, we opted for a non-particulate injectate consisting of 5% dextrose combined with vitamin B12. The 5% dextrose serves a dual purpose: it mechanically hydro-dissects the compressed nerve root while also providing analgesic effects [7]. Meanwhile, vitamin B12 has been shown to support neural regeneration, adding a therapeutic benefit to the injection [6].

## Conclusions

Our novel US-guided approach for cervical transforaminal injections was able to achieve accurate needle placement and successful epidural spread. We believe this approach could decrease radiation exposure for both clinicians and patients, optimize needle placement, and potentially improve patient safety and therapeutic outcomes.

## References

[1] Radhakrishnan K, Litchy WJ, O'Fallon WM, Kurland LT (1994). Epidemiology of cervical radiculopathy. A population-based study from Rochester, Minnesota, 1976 through 1990. Brain.

[2] Mansfield M, Smith T, Spahr N, Thacker M (2020). Cervical spine radiculopathy epidemiology: a systematic review. Musculoskeletal Care.

[3] Kose HC, Guven Kose S, Celikel F, Tulgar S, Akkaya OT (2024). Ultrasound-guided cervical selective nerve root block versus fluoroscopy-guided interlaminar epidural injection for cervical radicular pain: a randomized, prospective, controlled study. J Pers Med.

[4] Yang D, Xu L, Hu Y, Xu W (2022). Diagnosis and treatment of cervical spondylotic radiculopathy using selective nerve root block (SNRB): where are we now?. Pain Ther.

[5] Feeley IH, Healy EF, Noel J, Kiely PJ, Murphy TM (2017). Particulate and non-particulate steroids in spinal epidurals: a systematic review and meta-analysis. Eur Spine J.

[6] Chen CH, Huang HY, Huang AP, Jaw FS, Chen MC, Lin CW, Wang SP (2023). Ultrasound-guided perineural vitamin B12 injection for brachial plexus injury: a preliminary study. Cell Transplant.

[7] Lam SK, Reeves KD, Cheng AL (2017). Transition from deep regional blocks toward deep nerve hydrodissection in the upper body and torso: method description and results from a retrospective chart review of the analgesic effect of 5% dextrose water as the primary hydrodissection injectate to enhance safety. Biomed Res Int.

[8] Narouze SN, Vydyanathan A, Kapural L, Sessler DI, Mekhail N (2009). Ultrasound-guided cervical selective nerve root block: a fluoroscopy-controlled feasibility study. Reg Anesth Pain Med.

[9] Yamauchi M, Suzuki D, Niiya T (2011). Ultrasound-guided cervical nerve root block: spread of solution and clinical effect. Pain Med.

[10] Chang Chien GC, Candido KD, Knezevic NN (2012). Digital subtraction angiography does not reliably prevent paraplegia associated with lumbar transforaminal epidural steroid injection. Pain Physician.

[11] Smuck M, Maxwell MD, Kennedy D, Rittenberg JD, Lansberg MG, Plastaras CT (2010). Utility of the anesthetic test dose to avoid catastrophic injury during cervical transforaminal epidural injections. Spine J.

[12] Bush K, Mandegaran R, Robinson E, Zavareh A (2020). The safety and efficiency of performing cervical transforaminal epidural steroid injections under fluoroscopic control on an ambulatory/outpatient basis. Eur Spine J.

[13] Rozin L, Rozin R, Koehler SA (2003). Death during transforaminal epidural steroid nerve root block (C7) due to perforation of the left vertebral artery. Am J Forensic Med Pathol.

[14] Hoang JK, Apostol MA, Kranz PG, Kilani RK, Taylor JN, Gray L, Lascola CD (2010). CT fluoroscopy-assisted cervical transforaminal steroid injection: tips, traps, and use of contrast material. AJR Am J Roentgenol.

[15] Ma L, Wang Y, Yao M, Huang B, Deng J, Wen H (2023). Evaluating the extent of ultrasound-guided cervical selective nerve root block in the lower cervical spine: evidence based on computed tomography images. J Pain Res.

[16] Wu J, Xu Y, Pu S, Zhou J, Lv Y, Li C, Du D (2021). US-guided transforaminal cervical nerve root block: a novel lateral in-plane approach. Pain Med.

[17] Kumar N, Gowda V (2008). Cervical foraminal selective nerve root block: a 'two-needle technique' with results. Eur Spine J.

[18] Huntoon MA (2005). Anatomy of the cervical intervertebral foramina: vulnerable arteries and ischemic neurologic injuries after transforaminal epidural injections. Pain.

[19] Lagemann GM, Yannes MP, Ghodadra A, Rothfus WE, Agarwal V (2016). CT-fluoroscopic cervical transforaminal epidural steroid injections: extraforaminal needle tip position decreases risk of intravascular injection. AJNR Am J Neuroradiol.

[20] Van Zundert J, Huntoon MA, van Kleef M (2009). Complications of transforaminal cervical epidural steroid injections. Spine (Phila Pa 1976).

